# Reasons for using e-cigarettes and support for e-cigarette regulations: Findings from the 2020 ITC Malaysia Survey

**DOI:** 10.18332/tid/146364

**Published:** 2022-03-31

**Authors:** Farizah Mohd Hairi, Kok Tiong Goh, Pete Driezen, Amer Siddiq Amer Nordin, Anne Yee, Nur Amani Ahmad Tajuddin, Siti Idayu Hasan, Mahmoud Danaee, Ina Sharyn Kamaludin, Susan C. Kaai, Mi Yan, Matthew Grey, Anne C. K. Quah, Mary E. Thompson, Geoffrey T. Fong

**Affiliations:** 1 Nicotine Addiction Research Group, University of Malaya Centre of Addiction Sciences, University of Malaya, Kuala Lumpur, Malaysia; 2 Department of Social and Preventive Medicine, Faculty of Medicine, University of Malaya, Kuala Lumpur, Malaysia; 3 Department of Psychology, University of Waterloo, Waterloo, Canada; 4 School of Public Health Sciences, University of Waterloo, Waterloo, Canada; 5 Department of Psychological Medicine, Faculty of Medicine, University of Malaya, Kuala Lumpur, Malaysia; 6 Department of Primary Care Medicine, Faculty of Medicine, University of Malaya, Kuala Lumpur, Malaysia; 7 Department of Statistics and Actuarial Science, University of Waterloo, Waterloo, Canada; 8 Ontario Institute for Cancer Research, Toronto, Canada

**Keywords:** e-cigarettes, Malaysia, vaping, reasons for vaping, regulations

## Abstract

**INTRODUCTION:**

Malaysia has the largest e-cigarette (EC) market in Southeast Asia, and it has been estimated that 17% of adult daily cigarette smokers also used ECs on a daily basis in 2020. However, few studies have examined the reasons people use ECs in Malaysia. This cross-sectional study of adult cigarette smokers from Malaysia assessed reasons for EC use and their support for key proposed EC regulations.

**METHODS:**

Data are from the 2020 International Tobacco Control (ITC) Malaysia Wave 1 Survey of adult (aged ≥18 years) smokers who reported that they used ECs at least monthly (N=459 out of 1047 smokers). Weighted analyses were conducted on EC users’ reasons for using ECs and their support for various EC regulations.

**RESULTS:**

Smokers who used ECs at least monthly were more likely to be male, aged 25–39 years, of Malay ethnicity, married, more highly educated, and living in Peninsular Malaysia. Smokers who used ECs daily reported using ECs to reduce the number of cigarettes smoked (91.3%), pleasant taste (90.1%), to quit smoking (87.9%), and enjoyment (87.5%). Smokers who used ECs less than daily reported using ECs for their pleasant taste (weekly 89.4%, monthly 87.5%), curiosity (weekly 79.5%, monthly 88.8%), being offered EC by someone (weekly 76.3%, monthly 81.6%), and to reduce the number of cigarettes smoked (weekly 76.2%, monthly 77.6%). Smokers who also used ECs were most likely to support EC regulations requiring a minimum purchasing age (88.3%) and limiting nicotine concentration (79.6%), and least likely to support regulations banning EC fruit and candy flavors (27.1%).

**CONCLUSIONS:**

The most prevalent reasons for using ECs in Malaysia are comparable to those of other ITC countries, including Canada, US, England, and Australia. An understanding of use patterns of ECs, especially their interaction with cigarettes, are important in developing evidence-based regulations in Malaysia.

## INTRODUCTION

Over the past decade, e-cigarettes (ECs) have changed the global tobacco and nicotine product market. The increase in the use of ECs has led to international debate on their costs and benefits among researchers, advocates, and governments. Studies estimating the prevalence of lifetime and current use of ECs in different Asian countries reveal similar patterns of use across these countries. For example, the prevalence of lifetime use of ECs ranged from 0.2% in Japan (2017), 2.2% to 2.7% in Taiwan (2014–2015), 2.3% in Hong Kong (2014), 2.9% in 14 cities in China (2013–2014), to 6.6% in South Korea (2013), and 11.9% in Malaysia (2016)^1-7^. Use in the past 30 days was less common^5-7^. However, patterns of use were similar such that lifetime use tended to be higher among males, younger people, and current cigarette smokers^3,5,6^. In 2019, 4.9% of Malaysians aged ≥15 years reported currently using ECs in the previous month^8^.

Malaysia is an important country to examine with respect to EC use. It has the largest EC market in Southeast Asia, where projected EC sales are expected to remain stable into the 2020s at US$260 million per year^9^. Studies conducted in 2011–2019 have found that 8–14% of Malaysian cigarette smokers use ECs at least monthly^7,10,11^. A more recent study conducted in 2020 found that 17% of daily cigarette smokers in Malaysia also used ECs daily^12^, a striking finding since only 12.8% of cigarette smokers in England used ECs daily in 2020^13^. In summary, these studies suggest that EC use in Malaysia is very high.

Limited studies relying on convenience samples of current adult EC users from Malaysia find that three-quarters of users reported using ECs to cut down or quit smoking, for health reasons, and because ECs are less harmful to others than cigarettes^14-16^. This study expands on that research by examining reasons for using ECs among a representative sample of adult Malaysian cigarette smokers who vape. It also examines the level of support for potential laws regulating ECs among Malaysians who vape who would be most affected by these regulations should they be enacted. Reasons for use and support for regulations were examined by frequency of EC use to determine whether reasons and support differed by frequency of use.

## METHODS

Data came from the 2020 (Wave 1) International Tobacco Control Malaysia (ITC MYS1) Survey, a cross-sectional survey of 1047 current, adult cigarette smokers aged ≥18 years who smoked at least 100 cigarettes in their lifetime and who currently smoked at least once a month^17,18^. The ITC MYS1 Survey was an online survey conducted from 5 February 2020 to 3 March 2020. Respondents were recruited from a Rakuten Insight web panel that was nationally representative of internet users in Malaysia. Respondents were randomly selected using quota sampling methods to ensure sampling targets were reached (970 male smokers and 100 female smokers)^17^. Sampling weights were computed and calibrated to estimated population sizes to correct for oversampling of female smokers^17,18^. All respondents provided informed consent before completing the online survey. A full description of the survey methods can be found elsewhere^17,18^.

Respondents were asked whether they ever used an EC, even once. Those who did were asked whether they currently used ECs: ‘daily’, ‘less than daily but at least once a week’, ‘less than weekly, but at least once a month’, ‘less than monthly’, or ‘not at all’. Smokers who reported using ECs at least monthly (n=459) were classified according to their frequency of EC use (daily, weekly, monthly). Smokers who did not use ECs were excluded from this analysis.

EC users were asked to report one or more reasons for using ECs from a list of 15 reasons (Table 1). EC users were also asked whether they supported or opposed each of five possible regulations for ECs: 1) requiring the same minimum age for buying ECs as for combustible cigarettes; 2) limiting the amount of nicotine allowed in ECs and/or e-liquid; 3) banning the use of ECs in places where smoking is already banned; 4) banning EC and e-liquid promotions (free samples, coupons, and price discounts); and 5) banning fruit and candy flavors. Respondents answering ‘support’ or ‘strongly support’ were classified as supporting the regulation while those answering ‘oppose’, ‘strongly oppose’, or ‘don't know’ where classified as not supporting the regulation. Weighted bivariate analyses compared reasons for use and support for regulations by frequency of EC use and exact 95% confidence intervals were estimated for all percentages^19^. Rao-Scott χ^2^ tests were used to examine the overall association between frequency of EC use and each outcome (i.e. all reasons for use and support for regulations). If the overall χ^2^ test was statistically significant (p<0.05), logistic regression was used to test differences in outcomes by frequency of EC use and a Bonferroni correction adjusted for multiple comparisons. The descriptive statistics of the sample were unweighted. All other analyses were weighted and conducted using the survey procedures using SAS software (Version 9.4, SAS Institute Inc., Cary, NC, USA).

**Table 1:** Reasons for using e-cigarettes (ECs) among adult Malaysian smokers who used ECs at least monthly in 2020 by frequency of EC use (N=459, weighted estimates*)

*Reason for using e-cigarettes*	*%*	*Daily (n=212) 95% CI*	*%*	*Weekly (n=177) 95% CI*	*%*	*Monthly (n=70) 95% CI*	*p^†^*
**Reduce amount smoked/to quit**							
Don’t have to give up smoking if replace some cigarettes with ECs	71.9	63.2–79.6	58.7	47.9–69.0	63.0	48.1–76.3	0.122
To cut down on number of cigarettes smoked^§^	91.3^a^	85.3–95.4	76.2^b^	67.0–83.8	77.6^b^	64.8–87.5	0.003
Help me quit cigarettes^§^	87.9^a^	82.4–92.3	60.0^b^	49.3–70.1	72.6^b^	58.5–84.0	<0.001
**Less harmful/social acceptability**							
Less harmful to health than cigarettes^§^	81.5^a^	74.0–87.7	62.7^b^	51.4–73.1	67.1^ab^	52.3–79.7	0.005
Less harmful to health of others around me^§^	83.4^a^	76.2–89.2	61.4^b^	50.5–71.5	74.3^ab^	61.0–85.0	<0.001
More acceptable than smoking cigarettes	78.1	70.5–84.6	67.4	57.2–76.5	65.7	51.4–78.1	0.105
Use ECs where cigarettes are banned	49.4	40.9–57.9	38.6	29.6–48.3	52.0	37.4–66.3	0.150
**EC characteristics**							
ECs taste good	90.1	83.9–94.5	89.4	81.5–94.7	87.5	76.0–94.8	0.877
Enjoy using ECs^§^	87.5^a^	80.5–92.7	69.7^b^	59.4–78.8	65.3^b^	50.1–78.4	0.001
**Social influences**							
I was curious	73.3	65.4–80.2	79.5	69.7–87.4	88.8	76.3–96.1	0.092
Someone offered me an EC	69.6	61.8–76.7	76.3	67.3–83.9	81.6	68.3–91.1	0.203
ECs make me look cool	62.5	54.1–70.4	57.6	47.0–67.8	48.0	33.7–62.5	0.224
**Other reasons**							
Save money using ECs instead of cigarettes^§^	73.2^a^	64.7–80.7	52.7^b^	42.1–63.1	71.6^ab^	57.4–83.3	0.003
Help me control appetite/weight	31.9	24.5–40.1	24.9	16.8–34.5	36.3	23.0–51.5	0.293
Health professional advised me to try them	35.3	27.3–43.9	20.8	13.4–30.0	30.3	17.6–45.8	0.059

* Weighted estimates from the 2020 ITC Malaysia Wave 1 Survey.

† p-value from a Rao-Scott χ² test for the association between frequency of e-cigarette use and each outcome.

§ Pairwise differences between e-cigarette frequency of use groups were tested using logistic regression. Groups sharing common superscript letters were not statistically different from one another at α=0.05 after controlling for multiple comparisons using a Bonferroni correction. Groups having different superscript letters were statistically different from one another after controlling for multiple comparisons.

a or b : Groups sharing common superscript letters were not statistically different from one another at α=0.05 after controlling for multiple comparisons using a Bonferroni correction.

ab : Groups having different superscript letters were statistically different from one another after controlling for multiple comparisons.

## RESULTS

Most respondents (n=1047) were male (90.2%, n=944), aged 25–39 years (60.6%, n=635), and married (55.4%, n=580). Most respondents were from Peninsular Malaysia (84.7%, n=887), half were of Malay ethnicity (52.2%, n=547), and one-third were highly educated (31.8%, n=333). Of 1047 smokers, 43.8% (n=459) used ECs in addition to combustible cigarettes. Of these, 46.2% (n=212) were daily users, 38.6% (n=177) were weekly users, and 15.3% (n=70) were monthly users. The remaining 588 smokers used ECs less than monthly, never used ECs, refused to respond, or responded ‘don't know’. These 588 respondents were excluded from weighted analysis.

Table 1 presents the reasons for using ECs among respondents who used ECs at least monthly in 2020 by frequency of EC use (n=459). Among daily users, the most common reasons for using EC were to cut down the number of cigarettes smoked (91.3%), the pleasant taste of ECs (90.1%), to help them stop smoking (87.9%), and for enjoyment (87.5%). Weekly users indicated pleasant taste (89.4%), curiosity (79.5%), being offered ECs by someone (76.3%), and to cut down on cigarettes smoked (76.2%), as their reasons for using ECs. Monthly users indicated curiosity (88.8%), pleasant taste (87.5%), being offered ECs by someone (81.6%), and to cut down on cigarettes smoked (77.6%), as their reasons for using ECs.

Six of the reasons for using ECs differed significantly by frequency of EC use. A significantly greater percentage of daily users than weekly or monthly users reported cutting down on combustible cigarettes as a reason for using ECs (91.3% of daily users vs 76.2% of weekly users and 77.6% of monthly users, both Bonferroni p<0.05). Other reasons that differed by frequency of use were helping them to quit smoking (87.9% vs 60.0% and 72.6%, both p<0.05), the belief that ECs are less harmful compared to combustible cigarette (81.5% vs 62.7%, p<0.01; and 67.1%, p=0.13), ECs are less harmful to others (83.4% vs 61.4%, p<0.001; and 74.3%, p=0.46), enjoyment (87.5% vs 69.7% and 65.3%, both p<0.01), and ECs help them save money (73.2% vs 52.7%, p<0.01; and 71.6%, p=1.00).

**Figure 1:**
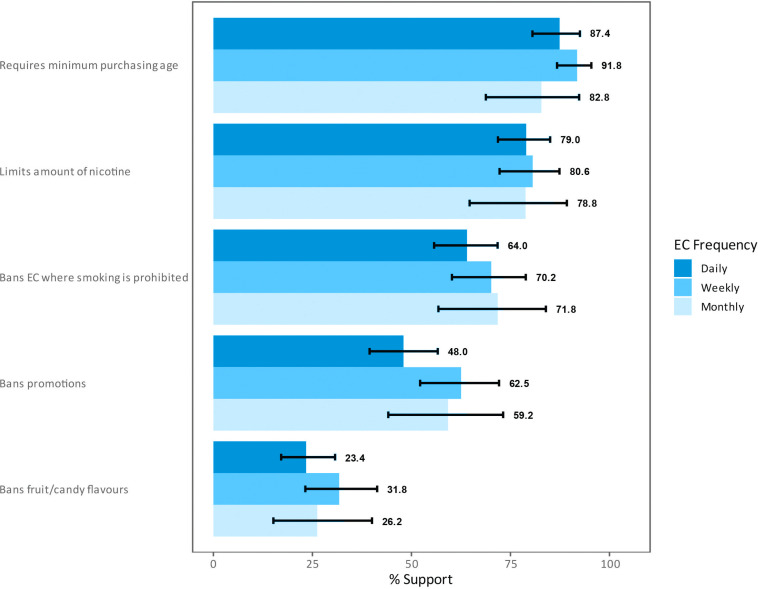
Support for EC and e-liquid regulations among adult Malaysian smokers who reported using EC at least monthly in 2020 by frequency of EC use (N=459, weighted estimates)

Figure 1 shows the level of support for EC and e-liquid regulations among smokers who used ECs at least monthly in 2020 by frequency of use. Irrespective of frequency of use, most smokers supported regulations requiring a minimum purchasing age (daily 87.4%, weekly 91.8%, monthly 82.8%), limiting nicotine content in ECs (daily 79.0%, weekly 80.6%, monthly 78.8%), and banning of EC use in smoke-free areas (daily 64.0%, weekly 70.2%, monthly 71.8%). The least supported regulation was banning fruit or candy flavors in ECs (daily 23.4%, weekly 31.8%, monthly 26.2%).

## DISCUSSION

Most Malaysian cigarette smokers who reported using ECs regularly reported using them: 1) to cut down on the number of cigarettes they smoked, 2) because they taste good, and 3) to help them quit smoking. Consistent with past studies from the US, Canada, England, and Australia^20,21^, two of the most common reasons for using ECs were to reduce cigarette smoking and to quit smoking. The importance of ECs to quit smoking was also found in three previous studies of Malaysian EC users^14-16^. Unlike those studies, this study examined reasons for using ECs by frequency of use. A greater percentage of daily users reported they used ECs to cut down on the number of cigarettes they smoked or to quit smoking completely than either weekly or monthly users. This suggests daily users might be more interested in using ECs to quit smoking than less frequent users. However, a greater percentage of daily EC users also reported they enjoyed using ECs than weekly or monthly users. Thus, more research is needed among Malaysian cigarette smokers who use ECs to better understand whether they use ECs because they enjoy them, because they use them to quit smoking, or whether enjoyment itself is related to using ECs to quit smoking.

While the import, sale, and use of ECs is permitted in Malaysia, the federal government wants to regulate ECs under the 1952 Poisons Act, which would classify ECs as a pharmaceutical product^9,22^. Although some Malaysian states have banned the sale of ECs (Penang, Kedah, Johor, Kelantan, Terengganu)^9^, there remains a need to understand how ECs should be regulated in Malaysia. These findings suggest there were no differences in support for regulations by frequency of EC use. However, among the regulations examined, requiring a minimum purchasing age of 18 years received the highest level of support, which is the current minimum age for purchasing combustible cigarettes in Malaysia. However, a regulation that would ban fruit or candy flavors in ECs was least supported by Malaysian smokers who also used ECs. This finding, in conjunction with the finding that taste was a reason for using ECs, points to the necessity of understanding how EC flavors influence EC use.

Unlike previous studies examining the reasons for EC use in Malaysia, this study was based on a sample of EC users that was representative of the Malaysian population of cigarette smokers.

### Limitations

Limitations include the use of self-reported data that may result in recall biases. In addition, the analysis was limited to the subset of respondents using ECs at least monthly. Some estimates (e.g. those for monthly EC users) are based on small sample sizes and may be less reliable, as suggested by the wide confidence intervals. Furthermore, these estimates were based on cigarette smokers only. Therefore, no conclusions can be drawn about reasons for use nor support for regulations among non-smokers who use ECs. Finally, respondents came from a panel that was representative of internet users in Malaysia, thus the study sample may be over-represented by younger, urban Malaysians who are more technologically knowledgeable than older, rural Malaysians.

## CONCLUSIONS

Among Malaysian cigarette smokers who also use ECs, the top three reported reasons for using ECs regularly were to reduce the number of cigarettes they smoke, to quit smoking, and taste. Most EC users supported policies that required a minimum purchasing age and limiting nicotine in EC; they were least likely to support banning EC flavors. The findings of this study, along with other studies on use patterns of ECs, particularly transitions to/from cigarettes and ECs over time, can provide guidance to policymakers to create evidence-based regulations of ECs in Malaysia and other countries.

## Data Availability

The data supporting this research are available from the authors on reasonable request.
